# Supplementary material for: Identification of an Upper Limit of Tumor Burden for Downstaging in Candidates with Hepatocellular Cancer Waiting for Liver Transplantation: A West–East Collaborative Effort
Source: Cancers (Basel). 2020 Feb 14;12(2):452. doi: 10.3390/cancers12020452 (PMC7072306; doi:10.3390/cancers12020452)
Supplement: Supplementary file 1 [file cancers-12-00452-s001.pdf]

Identification of an Upper Limit of Tumor Burden for Downstaging in Candidates with Hepatocellular Cancer Waiting for Liver Transplantation: A West–East Collaborative Effort

Quirino Lai, Alessandro Vitale, Karim Halazun, Samuele Iesari, André Viveiros, Prashant Bhangui, Gianluca Mennini, Tiffany Wong, Shinji Uemoto, Chih-Che Lin, Jens Mittler, Toru Ikegami, Yang Zhe, Shu-Sen Zheng, Yuji Soejima, Maria Hoppe-Lotichius, Chao-Long Chen, Toshimi Kaido, Chung Mau Lo, Massimo Rossi, Arvinder Singh Soin, Armin Finkenstedt, Jean C. Emond, Umberto Cillo and Jan Lerut

Additive Data

Participating centres

Data were obtained from twelve collaborative centres coming from United States, Europe, and Asia. The participating centres with the corresponding number of patients enrolled in the present study were: Padua (Italy; n = 627), New York Columbia University/Weill Cornell Medical Center (USA; n = 356), Brussels (Belgium; n = 296), New Delhi (India; n = 269), Innsbruck (Austria; n = 264), Hong Kong (SAR of People's Republic of China; n=260), Kyoto (Japan; n = 222), Rome Sapienza University (Italy; n = 197), Taiwan (Republic of China; n = 185), Mainz (Germany; n = 173), Kyushu (Japan; n = 149), and Hangzhou Shulan Health Hospital/First Affiliated Hospital (People's Republic of China; n = 93).

Population splitting in Derivation and Validation Set

The entire population of 3,091 patients was split into a Derivation Set of 2,318 candidates (75.0%) and a Validation Set of 773 candidates (25.0%). A block randomization was performed to maintain a similar representation of Western and Eastern centres in the two data sets. The entire population was divided in twelve subgroups ("blocks"), each one corresponding to every centre participating in the study. Then, patients within each block were randomly assigned to the Derivation or the Validation Set using a casual number generator. We arbitrarily decided to use the 75.0% and the 25.0% of the entire population for constructing the two different sets.

Statistical construction of the competing risk models

Typically, the studies based on competing-risk analyses deal with two competing risks [1,2]. In our model, three different competing risks were taken into account. Basically, HCC candidates can experience: 1) a post-waiting-list registration delisting due to worsening of liver function ("too sick or death") or tumour progression beyond transplantability criteria; if transplanted, recipients can experience: 2) a "tumour-specific" death, defined as post-transplant HCC recurrence unequivocally determining patient's death through disseminated intra- or extra-hepatic diffusion and loss of liver function due to tumour spread; and 3) a "non-tumour-specific death", defined as death after LT from any other, non-tumoral, cause.

In the Article, we specifically investigated the model for the risk of being transplanted and then die for HCC. We here reported the multivariable Fine & Grey models for delisting and for dying due to HCC-related and unrelated causes.

Competing events	SHR (95%CI)	p-value
**Delisting**		
Age at entry (per year)	1.049 (1.031–1.068)	0.001
MELD at entry (per unit)	1.033 (1.012–1.056)	0.002
Receive LDLT	0.422 (0.297–0.599)	0.001

Receive LT & die for other causes		
Age at entry (per year)	1.021 (1.005–1.036)	0.009
HCV positivity	1.309 (1.058–1.619)	0.013
MELD at entry (per unit)	1.022 (1.004–1.041)	0.012

Abbreviations: SHR, sub-hazard ratios; CI, confidence intervals; MELD, model for end-stage liver disease; LDLT, living donor liver transplantation; HCV, hepatitis C virus.

"Recalibration" of the upper acceptable limit for the risk of post-transplant HCC-related death

In our study, we "recalibrated" the coefficients proposed by Mazzaferro in the Metroticket 2.0 study[1] with the intent to design a model able to predict the risk of death in an intention-to-treat fashion. The WE-DS Model was created with a competing-risk analysis, namely fulfilling the same methodology proposed in the present Metroticket 2.0 study [1]. Moreover, we investigated the same competing event of Metroticket 2.0, namely the risk of dying due to recurrence after transplant. The only difference was that we calculated this risk from the time of first referral (intention-to-treat setting) instead of analysing it at LT time.

Interestingly, Metroticket 2.0 study provided un "a priori" upper limit of acceptability for five-year post-transplant HCC-related death, placing this value at 30% [1]. In other terms, using the cut-offs proposed by Mazzaferro, the Metroticket 2.0 score identified un upper limit of transplantability at LT time according to the combination of alpha-fetoprotein (AFP), number of nodules and diameter of the largest lesion.

We "recalibrated" this risk starting from a different time-point, namely from the time of first referral. We adopted different steps:

a) We calculated the risk of five-year post-transplant HCC-related death for each patient with the Metroticket 2.0 value predicted at liver transplantation. For calculating this risk, we used the original formulae coming from the Metroticket 2.0 study [1]:

0.8168342*log10AFP+0.226811*(Tumour number + Diameter in cm)

b) WE-DS predicted at first referral was recalculated in each patient for the same risk, using the following equation:

0.588989*log10AFP+0.142158*(Tumour number + Diameter in cm)

c) We matched in a plot the patients of the Derivation Set, obtaining the Figure 3A. We observed that the Metroticket 2.0 value at LT=30% corresponded to the value of WE-DS at first referral = 13%.

d) We recalibrated the risk at 13%, generating a new upper limit of tumor burden for downstaging as clarified in Figure 3B. In other terms, the upper achievable threshold to limit the risk of post-transplant HCC-related death to 13% was:

AFP 1-20 ng/mL and up to twelve lesions;
AFP=21-200 ng/mL and up to ten lesions;
AFP=201-500 ng/mL and up to seven lesions;
AFP=501-1,000 ng/mL and up to five lesions.

Table S1. Patients out of Milan Criteria and Up-to-Seven Criteria at first referral and liver transplantation in the different centres.

Centres	N of cases	MC OUT		UP-TO-7 OUT	
		Referral	LT	Referral	LT
**Brussels**	296	68	25	37	12
**Sapienza Rome**	197	45	27	16	8
**New Delhi**	269	132	143	90	96
**Kyushu**	149	38	38	22	23
**New York**	356	74	91	34	46
**Innsbruck**	264	85	54	49	36
**Taiwan**	185	61	58	29	23
**Mainz**	173	104	75	89	60

**Guangzhou**	93	60	61	50	50
**Kyoto**	222	77	77	62	62
**Hong Kong**	260	47	51	10	13
**Padua**	627	174	120	72	54

References

1. Mazzaferro, V.; Sposito, C.; Zhou, J.; et al. Metroticket 2.0 model for analysis of competing risks of death after liver transplantation for hepatocellular carcinoma. *Gastroenterology* **2018**, *154*, 128–139. DOI: 10.1053/j.gastro.2017.09.025
2. Berry, K.; Ioannou, G.N. Comparison of Liver Transplant-Related Survival Benefit in Patients With Versus Without Hepatocellular Carcinoma in the United States. *Gastroenterology* **2015**, *149*, 669–80. DOI: 10.1053/j.gastro.2015.05.025
